# Sociodemographic determinants of attrition in the HIV continuum of care in Brazil, in 2016

**DOI:** 10.1097/MD.0000000000009857

**Published:** 2018-05-25

**Authors:** Ana Roberta Pati Pascom, Mariana Veloso Meireles, Adele Schwartz Benzaken

**Affiliations:** Department of STI, AIDS and Viral Hepatitis, Ministry of Health, Brasília, Brazil.

**Keywords:** adherence, antiretroviral treatment, Brazil, HIV care continuum, HIV/AIDS, late diagnosis

## Abstract

The aim of this study was to identify sociodemographic factors associated with attrition in the 3 steps of the HIV continuum of care related to the 90-90-90 targets – access to diagnosis, treatment initiation, and virologic suppression, in Brazilian adults (15 years or older), in 2016.

Programmatic data were obtained from 2 information systems from the Brazilian Ministry of Health, which register all antiretroviral therapy (ART) dispensations and all CD4^+^ and viral load counts (VL) performed within the country's public health system. The 3 attrition indicators were late presentation to care, defined as a first CD4 count <350 cells/mm^3^ among ART-naive individuals who performed a first CD4^+^ count in 2016; not being on ART, defined as having no recorded dispensation within the last 100 days of the year, among those who were linked to care in 2016; and not being virologically suppressed, defined as having the last recorded VL >200 copies/mL in 2016, among those with a recorded VL count who were on treatment for at least 6 months. Association of sociodemographic factors with these indicators was analyzed by unconditional logistic regression analysis.

Lower educational level and black/brown/indigenous race/color were associated with worse outcomes in the 3 indicators. Environmental indicators, namely the region, size, and social vulnerability index of the municipality of residence, also played an important role in the models. Younger age was strongly associated with not being on ART and not showing virological suppression.

Our findings help identify the barriers in the different stages of the HIV continuum of care, which need to be addressed in order to progress toward the achievement of the 90-90-90 targets.

## 1 Introduction

The HIV continuum of care refers to the “sequential steps that take individuals from HIV diagnosis through enrollment into care, timely initiation of antiretroviral therapy (ART), adherence to treatment, and viral suppression.”^[1]^ It has become a key tool for the quantitative monitoring and evaluation of HIV care and treatment policies.^[2]^ In fact, in recognition of the continuum of care's crucial role and the need to scale up efforts to the end the HIV/AIDS epidemic, the Joint United Nations Program on HIV/AIDS (UNAIDS) adopted the ambitious 90-90-90 targets, which state that, by 2020: 90% of all people living with HIV (PLWH) should know their HIV status; 90% of all people with diagnosed HIV infection should be on ART; and 90% of all people receiving ART should have achieved viral suppression.^[3]^

Brazil was one of the first countries to recommend treatment for all regardless of CD4^+^ cell counts and to embrace the 90-90-90 targets, including its indicators in the country's HIV clinical monitoring system.^[4,5]^ Estimated HIV prevalence in Brazil was 0.4% in 2016, representing approximately 830,000 PLWH, of whom 84% (694,000) had been diagnosed, 72% (498,000) of the diagnosed were receiving ART, and 91% (452,000) of those on ART had achieved virological suppression - viral load (VL) < 1000 copies/mL.^[5]^ To strengthen the response to the HIV epidemic, it is important not only to identify the attrition along each step of the cascade but also to understand the factors that interfere in these drop-outs such as those that refer to the health care system, to individual (behavioral and biologic) characteristics, and to stigma and discrimination.^[6,7]^ In the present analysis, we aim to identify sociodemographic factors associated with attrition within the 3 steps of the continuum that refer to the 90-90-90 targets: access to diagnosis, initiation of treatment, and virological suppression in Brazilian adults (15 years old or over), in 2016.

## 2 Methods

The detailed method for estimation of the HIV continuum of care by the Brazilian AIDS Program is described elsewhere,^[8]^ and in this study, we present a brief outline of the definitions and information systems used.

### 2.1 Data sources/Information systems

Established in 2000, Brazil's Antiretroviral Medication Logistics Control System (Sistema de Controle Logístico de Medicamentos Antirretrovirais/SICLOM) documents all antiretroviral (ARV) dispensations in the country. Currently, in over 95% of the dispensing centers of the country, information is collected with individual-level data on the patients. In Brazil, all ARV have been available free of charge since 1996,^[9]^ and they are not sold in drugstores. Thus, the system captures virtually all PLWH in treatment in the country.

Established in 2006, Brazil's Laboratory Test Control System (Sistema de Controle de Exames Laboratoriais/SISCEL) gathers information about all CD4^+^ and VL counts performed within the country's the Unified Health System (SUS), its public health system. SISCEL does not capture tests performed in private laboratories, typically accessed by people who have access to private health care plans, estimated at 25.4% in 2016.^[10]^

These 2 systems are managed by the Brazilian AIDS Program and share a common registration database, which uses a unique identifier for each patient. De-duplication is conducted monthly by means of a deterministic process, using the patient's name, his or her mother's name, municipality of birth, and CPF (Brazil's individual taxpayer registration number).

### 2.2 Indicators

#### 2.2.1 Diagnosis

To estimate the total number of PLWH in Brazil in 2016, the AIDS Program used the Spectrum software^[11]^; therefore, no individual-level data are available to directly assess the attrition between HIV infection and diagnosis. In this analysis, therefore, we chose the surrogate indicator of late presentation, as patients who present late are typically those who remained infected for longer periods before being diagnosed. Late presentation was defined as a first recorded CD4^+^ count in ART-naive patients below 350 cells/μL.

#### 2.2.2 Treatment

The definition for being on treatment in a certain year is having a recorded ART pick-up within the last 100 days of that same year. The Brazilian AIDS Program adopts this definition, as ART prescriptions in the country are typically made for 30 days, extendable for a maximum of 90 days. Attrition in access to treatment was estimated relative to all PLWH who were considered linked in 2016, namely, those who had at least 1 CD4^+^ or VL count or an ART pick-up during that year.

#### 2.2.3 Virological suppression

Attrition for virological suppression was defined as last VL count recorded in 2016 above 200 cells/mm^3^ in those who were on treatment by the end of the year (i.e., at least 1 ART pick-up in the last 100 days) for at least 6 months and had carried out at least 1 VL count in the year.

**Figure 1:**
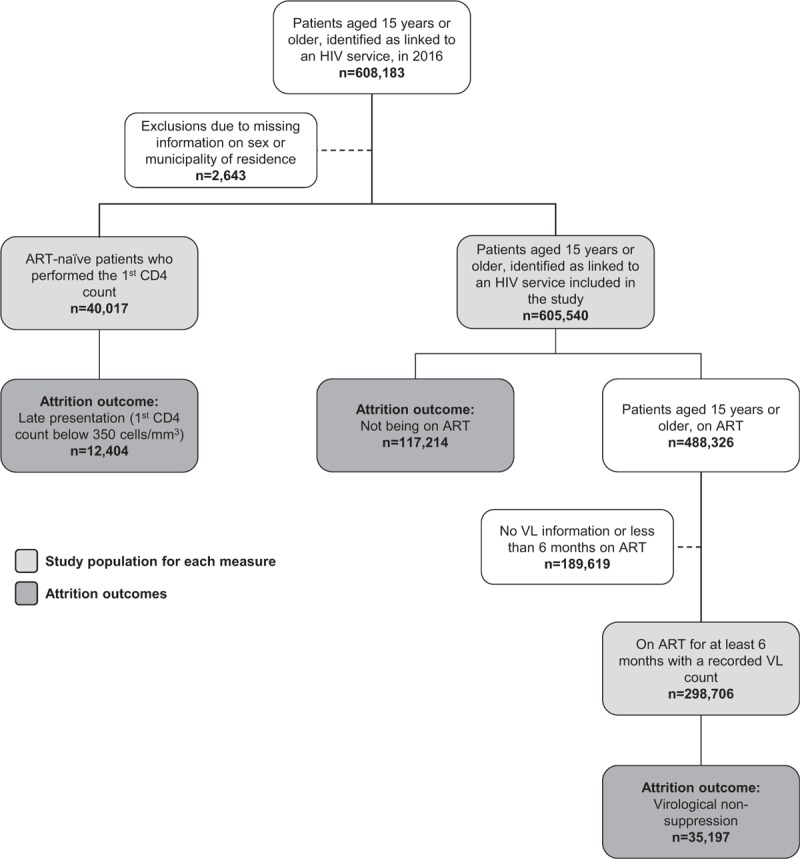
Study population flowchart.

All indicators were analyzed only for adults aged 15 years or older. We excluded records that had unknown sex or municipality of residence. The study population for each of the indicators is presented in Fig. 1.

### 2.3 Statistical analyses

The independent variables we assessed were sex; age (15–24 and over 25 years), race/color (white/yellow, black/brown/indigenous and unknown); schooling (0–7 and 8+ years); region of residence (North, Northeast, Center-West, Southeast and South of Brazil); size of municipality (less than 100,000 and 100,000+ inhabitants); and social vulnerability index – SVI – (very low/low, medium, and high/very high). The SVI, developed by the Brazil's Institute for Applied Economic Research, presents information on 3 dimensions that correspond to “sets of assets, resources or structures, whose access, absence or insufficiency indicate that the standard of living of families is low, suggesting, in the limit, non-access and non-compliance to social rights.”^[12]^ These dimensions are urban infrastructure (including indicators of access to basic sanitation services and urban mobility), human capital (including health and education indicators), and income and labor (including indicators of *per capita* income, occupation, and type of employment relationship). The index is presented on a 0 to 1 scale, in which 1 is the maximum possible social vulnerability.

We used unconditional univariable and multivariable logistic regression models to identify which factors were associated with the 3 attrition measures. For the late presentation indicator – due to different profiles regarding the diagnosis of men and women – we developed 2 separate models, one for each sex. We determined crude and adjusted odds ratios (OR and aOR) with respective 95% confidence intervals (95% CIs). Eligible variables for the multivariable analysis were identified in the crude analysis by presenting a hypothesis test with a *P* value <.20. The final model was selected on the basis of backwards selection considering a *P* value ≤.05 as the cutoff point for statistical significance.

### 2.4 Ethics

All data used in this paper were obtained according to guidelines from the Health Surveillance Secretary of the Ministry of Health in Brazil. We ensured data confidentiality by using anonymized databases, after creating unique personal identifiers. Furthermore, data analysis was performed by authors who are responsible for maintaining the VL, CD4, and ART databases in the Brazilian Ministry of Health.

## 3 Results

Figure 1 presents the study populations for each of the models and the frequencies of the outcomes. Late presentation for care was found in 44.0% (12,404/40,017) of the ART patients included in the analysis; among those linked in 2016, 19.4% (117,214/605,540) were not on ART by the end of the year; and among those on treatment for at least 6 months with a recorded VL count, 11.8% (35,197/298,706) did not achieve virological suppression (VL < 200 copies/mL). Table 1 presents the distribution of the independent variables for each of the models, and Tables 2 and 3 present the results of the univariable and multivariable logistic regression models.

**Table 1:**
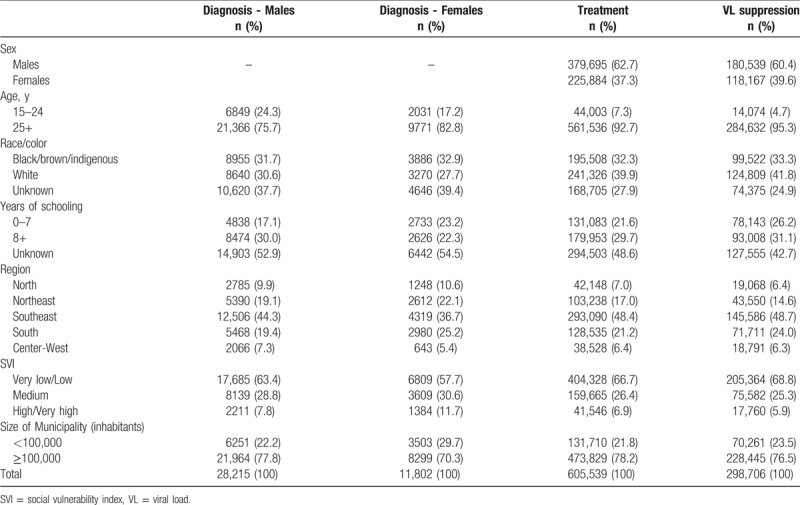
Distribution of sociodemographic characteristics of PLHIV included in each of the analyses.

**Table 2:**
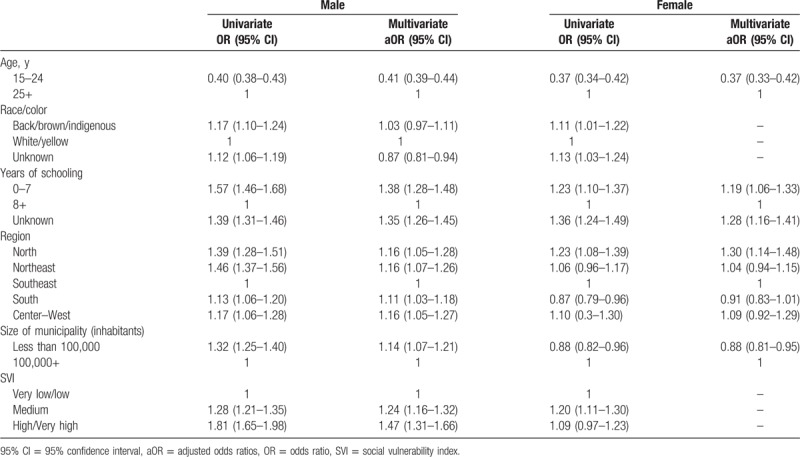
Results from the univariate and multivariate unconditional logistic regression models for late presentation (CD4 <350 cells/mm^3^).

**Table 3:**
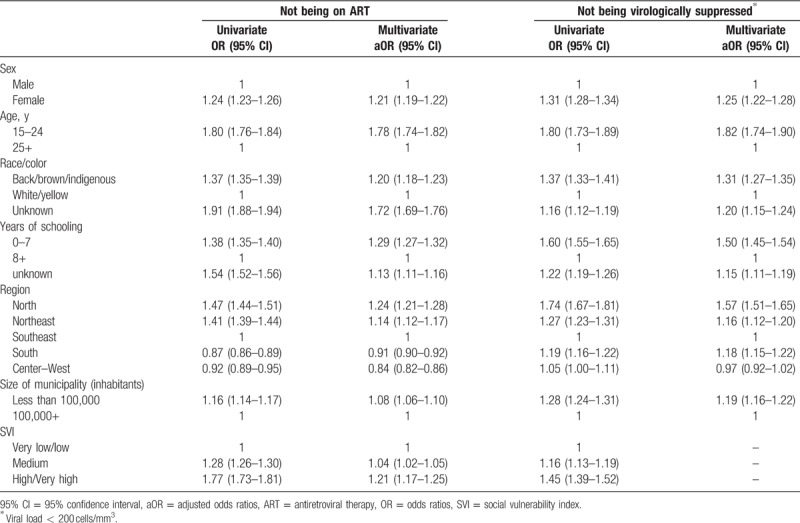
Results from the univariate and multivariate unconditional logistic regression models for not being on ART (among those linked) and not being virologically suppressed^∗^ (among those on ART).

### 3.1 Late presentation for care

In the univariable analysis, all variables were statistically associated with the odds of presenting late to care, in both males (all *P* < .001) and females (*P* race/color = .02; *P* size of municipality = .003; all other *P* < .001).

In the adjusted analyses, lower educational level in both men and women was associated with higher odds of presenting late (males aOR 1.38, 95% CI 1.28–1.48 and females aOR 1.19, 95% CI 1.06–1.33 0–7 years of schooling relative to 8+ years). In males, odds of late presentation were higher in municipalities with higher SVI (aOR 1.47, 95% CI 1.31–1.66 for high/very high and aOR 1.24, 95% CI 1.16–1.32 for medium, relative to very low/low SVI) and located in regions other than the Southeast. In females, living in the North region increased the odds of presenting late to care by 30% (95% CI 1.14–1.48). Size of the municipality also presented different results among the sexes; living in smaller municipalities was protective for women (aOR 0.88, 95% CI 0.81–0.95), while the opposite was observed in men (aOR 1.14, 95% CI 1.07–1.21).

### 3.2 Access to ART

All variables analyzed were significantly associated with being linked to the health services and not being on ART, both in the univariable (all *P* < .001) and multivariable analysis (all *P* < .001) (Table 3). Being female (aOR 1.21, 95% CI 1.19–1.22), black/brown/indigenous (aOR 1.20, 95% CI 1.18–1.23), having 0 to 7 years of schooling (aOR = 1.29, 95% CI 1.27–1.32), living in the North region (aOR 1.24, 95% CI 1.21–1.28), and in municipalities with high/very high social vulnerability index (aOR 1.21, 95% CI 1.17–1.25) increased in more than 20% the odds of not being on ART. Younger patients showed a 78% (95% CI 1.74–1.82) higher odds of not being on ART. In contrast, living in the South (aOR = 0.91, 95% CI 0.90–0.92) or Center-West (aOR = 0.84, 95% CI 0.82–0.86) was inversely associated with the odds of not being on ART.

### 3.3 Virological suppression

Logistic regression revealed that all variables were statistically associated with the odds of not being virologically suppressed in the univariable analysis (all *P* < .001) (Table 3). In the adjusted analysis, younger age increased the odds of nonvirological suppression by 82% (95% CI 1.74–1.90). Having 0 to 7 years of schooling (aOR = 1.50, 95% CI 1.45–1.54) and living in the North region (aOR = 1.57, 95% CI 1.51–1.65) increased by more than half the odds of not being virologically suppressed, while being female and black/brown/indigenous raised the odds of not being virologically suppressed by 25% (95% CI 1.22–1.28) and 31% (95% CI 1.27–1.35), respectively.

## 4 Discussion

We sought to examine the importance of certain sociodemographic and environmental characteristics to the attrition in the most important steps of the continuum of care in Brazil, in 2016. Some of our most important findings were the strong association of younger age with not being on treatment or virologically suppressed, and the effect of variables well known to their relationship with poverty,^[13]^ such as race/color, schooling, and SVI, on the 3 attrition measures.

Age was significant in all models analyzed. For diagnosis only, young people aged 15 to 24 years presented better results than individuals aged 25 years and over. This finding, however, probably stems from the fact that infection diagnosed in young people is generally recent, as the average age of onset of sexual activity is 16 years ^[14]^ and the most frequent source of infection is sexual transmission.^[15]^ Studies have shown that HIV testing coverage in Brazil is lower among young people.^[14,16]^ In addition, late diagnosis gradually increases with age, suggesting that a relevant part of positive cases found in the older age groups are infections acquired in youth.^[5,17]^ Of note, younger ages increased attrition in both access to ART and in virological suppression by around 80%. This finding, which is consistent with other studies,^[18,19]^ suggests that younger patients not only have lower ART coverage but also present poorer levels of adherence, highlighting the need to roll out interventions that are specifically designed for this group.

CD4 levels at presentation to care is the only indicator in which women performed better than men. Antenatal care is a common route of access for HIV diagnosis in Brazil,^[20,21]^ as HIV testing is mandatory during pregnancy in SUS, with a 95% estimated coverage.^[22]^ Access to antenatal care is higher in smaller municipalities,^[22]^ which is a possible explanation for the better results relative to late presentation found in women living in locations with less than 100,000 inhabitants. On the contrary, this variable was associated with worse outcomes in the 3 other measures, late presentation in men, access to ART, and virological suppression, which can probably be explained by the concentration and higher quality of specialized HIV care services in big cities.^[23]^ These findings reinforce the need to pursue decentralization of HIV care to primary health care units, as these are present in 5423 out of the 5560 Brazilian municipalities.^[24]^ This process has been initiated in Brazil over the last few years and should be expanded.

Results of the multivariable analyses of access to ART and virological suppression were very similar, with the exception of SVI, which was only significant for access to ART. Individual characteristics of PLWH, such as being female, younger than 25 years old, of black/brown/indigenous race/color, and having lower levels of education decrease the odds both of accessing ART and achieving virological suppression. In addition, structural-level factors are also associated with the study's measures, for instance the region and size of municipality of residence. Poorer outcomes observed in the North region, where the Amazon rain forest is located, are most likely related to the scattered population, deficient infrastructure, and difficulties related to geographical access. To achieve better results for the HIV epidemic in this region, these barriers should be addressed using innovative interventions, such as point-of-care HIV testing and VL counts.^[25,26]^

This study presents some limitations inherent to the secondary sources of information used. The fact that only public laboratories report CD4^+^ and VL results to the laboratory system limits the generalizability of our findings to patients followed in the public health system, especially for the late presentation and virological suppression measures. In addition, the information systems used do not include variables potentially relevant to the outcomes, such as gender identity, sexual orientation, drug use, and sex work. Furthermore, we acknowledge the possibility of ecological fallacy related to the use of SVI and size of municipality. However, we chose to include these variables in order to account for structural factors that are known sources of inequality in health care access in Brazil. We chose bearing this risk of bias over including exclusively individual-level demographic variables.

Our findings suggest that not only individual characteristics but also the living conditions in the patients’ place of residence greatly affect access to HIV services. The similarity between the results of the treatment and virological suppression measures reveal that, despite being distinct stages of care, the barriers to be overcome in order to improve these indicators are similar. Thus, in the development of HIV-related public policies, it is of paramount importance to consider, in addition to specific scientific evidence, the well-known health inequities present in the country.^[27]^ The achievement of the 90-90-90 targets is contingent upon the focus on intersectoral initiatives for the improvement of the timely access to HIV diagnosis and treatment and of adherence levels.

## Author contributions

**Conceptualization:** A.R.P. Pascom, M. Meireles, A.S. Benzaken.

**Data curation:** A.R.P. Pascom, M. Meireles.

**Formal analysis:** A.R.P. Pascom, M. Meireles.

**Methodology:** A.R.P. Pascom.

**Software:** A.R.P. Pascom, M. Meireles.

**Writing – original draft:** A.R.P. Pascom, M. Meireles.

**Writing – review & editing:** A.R.P. Pascom, M. Meireles, A.S. Benzaken.
